# Roles of the FGF-FGFR Signaling System in Cancer Development and Inflammation

**DOI:** 10.3390/cells10092231

**Published:** 2021-08-28

**Authors:** Antoni Wiedlocha, Ellen Margrethe Haugsten, Malgorzata Zakrzewska

**Affiliations:** 1 Department of Molecular Cell Biology, Institute for Cancer Research, The Norwegian Radium Hospital, Oslo University Hospital, Montebello, 0379 Oslo, Norway; 2 Center for Cancer Cell Reprogramming, Institute of Clinical Medicine, Faculty of Medicine, University of Oslo, Montebello, 0379 Oslo, Norway; 3 Department of Tumor Biology, Institute for Cancer Research, The Norwegian Radium Hospital, Oslo University Hospital, Montebello, 0379 Oslo, Norway; 4 Department of Protein Engineering, Faculty of Biotechnology, University of Wroclaw, Joliot-Curie 14a, 50-383 Wroclaw, Poland

For multi-cellular organisms to organize tissues, their cells must communicate with each other. This communication occurs through a number of regulatory proteins, among which growth factors have an important function. Growth factors produced within a cell are secreted outside the cell to convey the instruction to neighboring cells in the tissue. Receiver cells present specific receptors at their surface that recognize and bind the secreted growth factors. Once a specific receptor at the cell membrane is occupied by a growth factor, a signal is transmitted to the cell interior. One such communication system consists of the fibroblast growth factors (FGFs) and their receptors (FGFRs) [1].

FGFs and their receptors have formed an important regulatory signaling system (operative in vertebrates and invertebrates) that most likely originated very early in animal evolution, as they were present in the common eumetazoan ancestor of Cnidaria and Bilateria [2,3]. In mammals, there are 22 members of the FGF family and four high affinity, cell surface localized FGF receptors (FGFR1–4) with tyrosine kinase activity. Most FGFs act in a paracrine or autocrine manner and are referred to as canonical FGFs. Canonical FGFs are subdivided into five subfamilies: FGF1/2, FGF4/5/6, FGF3/7/10/22, FGF8/17/18 and FGF9/16/20. Their affinity for heparan sulfate proteoglycans (HSPGs), and thus association with the extracellular matrix, underlies the local nature of their activity. HSPGs also protect canonical FGFs from extracellular proteases and are required as co-receptors for stable binding between FGFs and FGFRs [4]. Some FGFs act as endocrine hormone-like or metabolic hormones, controlling metabolic homeostasis of the organism and are specific to vertebrates. These include members of the FGF19/21/23 subfamily. Unlike canonical FGFs, these FGFs have a reduced affinity for HSPGs, allowing them to reach the blood stream as well as travel to and bind to target cells in various organs. Endocrine FGFs require the presence of additional co-receptors, Klotho proteins (αKlotho or βKlotho), to form active, stable FGF/FGFR complexes [5]. The FGF subfamily, FGF11/12/13/14, consists of intracrine members of the family that are involved in neuronal development and affect the activity of voltage-gated channels [1,6]. 

The high affinity, cell surface FGF receptors belong to a family of receptor tyrosine kinases (RTKs). Their intracellular tyrosine kinase domain is activated upon ligand binding. For three of the four high-affinity receptors (FGFR1–3), there are a number of splicing variants, which are characterized by different cellular expression. Active receptors induce various intracellular, downstream signaling pathways leading to positive or negative regulation of cell differentiation, proliferation, migration and survival. The different biological responses induced by the FGF/FGFR signaling system seem to depend on the receiver cell type [1,4].

The FGF-FGFR interaction is particularly important during development, as FGF-FGFR signaling is a key regulator of mesenchymal–epithelial communication and is essential for organogenesis [4]. Furthermore, FGF-FGFR signaling also plays crucial roles in tissue homeostasis, including tissue repair and regeneration after trauma [7]. Therefore, many pathological conditions, such as different types of developmental and metabolic disorders, as well as malignant diseases, are caused by impaired FGF-FGFR signaling [1,4,8].

The main objective of this Special Issue of *Cells* is to highlight the latest advances in understanding the role of the FGF-FGFR signaling system in the development of neoplastic diseases and in the induction and maintenance of inflammation and its sequelae. The Special Issue contains 13 articles consisting of 3 original articles and 10 review articles that cover a broad spectrum of the involvement of the FGFs-FGFRs in malignant transformation and inflammation. Various aspects of FGF-FGFR signaling and downstream partners in several types of cancer such as breast cancer [9,10], pancreatic cancer [11], GIST and soft tissue sarcomas [12] are discussed. In addition, more general mechanisms of FGF-FGFR signaling in cancer including negative regulation of signaling [13], noncanonical FGF/FGFR binding partners in cancer [14] and role of oncogenic fusion proteins in centrosome and cilia formation [15] are presented. An important part of the Special Issue is dedicated to the therapeutic potential of targeting of FGF/FGFRs [16,17] and downstream signaling partners [9,18] in cancer and inflammatory diseases.

In the first original paper, Vikan A.K. et al. [19] combined a photochemical internalization (PCI) approach with the delivery of FGF2 conjugated to a highly cytotoxic ribosome-inactivating protein, saporin (FGF-SAP), to test the efficacy and specificity of the drug towards cells overexpressing FGFRs. PCI is a light-controlled method releasing endocytosed material into the cytosol. The efficacy and selectivity of PCI for FGF-SAP was evaluated in osteosarcoma cells overexpressing FGFR1 and the authors demonstrated that PCI greatly enhances cytotoxicity of the conjugate showing effective cell killing at pM concentration. However, the specificity towards cells with high FGFR expression was compromised because FGF-SAP also undergoes endocytosis in cells with low/no levels of FGFRs through binding to low affinity receptors. HSPGs present at the cell surface of all cells. The authors propose that in the next generations of FGF-toxin conjugates, the HSPG binding site in FGFs should be mutated, to avoid uptake through this pathway.

The second original work, published in this issue by Rajendran R. et al. [17], demonstrates the relevance of FGF-FGFR signaling pathways in the pathogenesis of multiple sclerosis (MS), a chronic inflammatory and neurodegenerative disease of the central nervous system (CNS). The authors showed that application of the FGFR-specific inhibitor, AZD4547, and dovitinib, which targets a broader spectrum of the tyrosine kinase receptors, disrupted the FGFR signaling cascade in oligodendrocytes in vitro. Disruption of FGF/FGFR signaling resulted in upregulation of BDNF/TrkB signaling and remyelination by increased myelin-specific protein production. Thus, FGFR inactivation may protect against further development of multiple sclerosis. 

Rajendran et al. [16] published a review article also on MS providing an update on the role of FGF/FGFR signaling pathways in the progression of this disease. A summary of the current knowledge leads the authors to conclude that FGFR inhibition may be a promising approach to regulate inflammation and induce neuroprotection in the CNS.

In another study, Boothyby-Schoemaker et al. [9] sought to elucidate the interplay between leptin, leptin receptor (LepR) and FGF2/FGFR1-induced signaling in primary human breast cancer associated with obesity. Human serum FGF2 levels are increased in obese individuals. Additionally, high serum levels of FGF2 have been associated with endocrine-resistant breast cancer. The authors demonstrate a significant relationship between LepR and FGFR1 in breast cancer tissue. A crosstalk between these two signaling pathways may occur through Jak2, consequently indicating that Jak2 could be a useful therapeutic target for breast cancer patients with visceral adiposity and high serum levels of FGF2 and leptin.

Nita et al. [15] reviewed the current literature concerning the relationship between FGFR signaling, primary cilia and pathological conditions, such as developmental ciliopathies and malignant diseases. The authors focus on oncogenic FGFR fusion proteins identified in cancer and their association with the centrosome cycle and consequently with mitotic spindle assembly and ciliogenesis. Finally, they speculate that restoring ciliogenesis or cilia function might be a promising new approach to attenuate tumor growth. Therefore, clinical studies evaluating cilia as a target for cancer therapy should be considered.

The dual role of FGF19/15 (FGF15 is the rodent form of FGF19) in the transplanted liver, either to repair the damaged liver or to promote tumorigenesis, described by Caballeria-Casals et al. [20] as a “Dr. Jekyll or Mr. Hyde” effect, remains an unsolved problem concerning risk factors for liver transplantation. The anti-apoptotic and pro-regenerative properties of FGF19/15 signaling in the liver are involved in (i) liver regeneration and (ii) also strongly participate in tumorigenic processes that often culminate in liver cancer. The authors summarize the current findings concerning FGF19/15 and liver tumorigenesis as well as FGF19/15 and liver regeneration. Furthermore, they discuss approaches to circumvent the pro-tumorigenic roles of FGF19/15 while maintaining its pro-regenerative properties and suggest that the development and use of non-tumorigenic variants of FGF19/15 may be a potential therapeutic option. 

The negative regulation of FGFR signaling is the subject of a review article by Szybowska et al. [13]. It should be noticed, that in contrast to the well-studied mechanisms of FGFR activation and signaling, the mechanisms mediating receptor deactivation are more poorly understood. The article summarizes the most recent literature on various mechanisms of negative regulation of activated FGFRs, such as endocytosis, phosphatase activity, negative regulatory proteins and negative feedback phosphorylation events. Deregulation of each of these mechanisms can lead to the development of highly malignant diseases or skeletal and metabolic disorders.

FGFR signaling can also be regulated by so-called noncanonical binding partners such as adhesion molecules and extracellular matrix components. The role of these noncanonical partners in FGFR signaling in cancer is reviewed by Ferguson et al. [14]. Direct interaction between FGFRs and cell adhesion molecules or extracellular matrix proteins can induce highly invasive, metastatic phenotypes of given cancer cells. Moreover, interactions of FGFRs with such noncanonical partners may also regulate angiogenesis and resistance to anti-cancer therapy. Thus, the authors suggest that noncanonical FGFR binding partners promote FGFR-induced tumorigenesis even in the absence of *FGFR* genetic aberrations.

In the review by Park T. [18], the roles of two signaling transducers, CT10 regulator of kinase (Crk) and Crk-like (CrkL) in development of human cancers are discussed. Crk and CrkL are cellular counterparts of the viral oncoprotein v-Crk, encoded by *v-crk* found in chicken tumor virus 10 and avian sarcoma virus. The author describes the molecular background of Crk and CrkL signaling and their involvement in cell transformation, tumor growth, migration and metastasis. They also indicate that Crk and CrkL may contribute to FGF/FGFR signaling in malignant tissue and suggest that these oncoproteins might be considered as molecular targets in cancer therapy. 

The Special Issue also contains review articles summarizing the current state of knowledge on the involvement of FGF/FGFR in specific forms of cancer, such as lung cancer [21], pancreatic cancer [11], sarcomas, [12] and breast cancer [10]. In some subgroups of lung cancer (small-cell lung cancer, non-small-cell lung cancer and squamous cell carcinoma) the incidence reaches 6–22%. In their review article, Pacini et al. [21] describe the various FGFR aberrations contributing to lung cancer progression. They also discuss the role of FGFRs in the acquisition of resistance to inhibitory therapies targeting EGFR, ALK, BRAF and KRAS. The authors emphasize the importance of developing additional robust biomarkers to select patients who benefit from FGFR-directed therapy to improve the clinical effectiveness. 

Carter et al. [11] review the role of FGFR signaling in the progression of pancreatic ductal adenocarcinoma (PDAC). PDAC is the most lethal form of pancreatic cancer and new specific diagnostic methods and more effective therapies are needed. This paper provides an update on the changes in FGF and FGFR in PDAC patients and in some of the most commonly used PDAC cell lines. The authors also highlight the role of FGFR signaling in the cross-talk between PDAC cells and the microenvironment as well as the crosstalk-between FGF/FGFR signaling and other signaling pathways such as Wnt/β-catenin, Sonic hedgehog and transforming growth factor β. In pre-clinical studies, specific FGFR inhibitors have shown promising effects in reducing tumor growth and bone metastasis and the authors consider FGFR as a potential molecular target in PDAC. 

Sarcomas are a rare but heterogenous group of malignances originating from mesenchymal tissue with a prevalence of around 1% of all cancers and 10–15% of pediatric cancers. These types of cancers have limited treatment options. Patients are usually treated with chemotherapy and/or radiotherapy in combination with surgery and would benefit from new personalized approaches. Napolitano et al. [12], in their review article, report on alterations of the FGFR signaling cascades identified in different types of sarcomas, mainly gastrointestinal stromal tumors, rhabdomyosarcomas and lipomas. Overall, approximately 4% of all sarcoma patients have targetable alterations in FGFRs. The authors also discuss the potential clinical use of FGFR inhibitors to treat these malignant diseases and highlight the need to develop biomarkers to better stratify patients who may benefit from such therapy. 

In their review on aberrant FGFR signaling in breast cancer, Sobhani et al. [10] focus on the role of FGFR signaling in CDK4/6 inhibitor-resistant disease. CDK4/6 inhibitors have been approved by FDA for the treatment of hormone receptor positive and epidermal growth factor receptor 2 negative breast cancer. Unfortunately, some patients develop resistance to these therapies. The authors describe the mechanisms of FGFR signaling during breast cancer development and discuss in detail why aberrant FGFR signaling may be responsible for the acquisition of resistance to CDK4/6 inhibitors. They postulate that targeting the FGFR pathway could overcome CDK4/6 inhibitor resistance during treatment of these subgroups of breast cancer. 

In conclusion, the Special Issue “Fibroblast Growth Factor Receptor (FGFR) Signaling Pathway in Cancer and Inflammation” summarizes the current state of knowledge on the role of the FGF/FGFR signaling system in the regulation of body homeostasis, with particular emphasis on the involvement of this signaling system in the development of various pathological conditions, mainly cancer. 
